# Perioperative effects of the quadratus lumborum block in percutaneous biliary drainage: a prospective randomized controlled study

**DOI:** 10.55730/1300-0144.6351

**Published:** 2026-06-01

**Authors:** Seyyid Furkan KINA, Can Ozan YAZAR, Cem Koray ÇATAROĞLU, Erdi TANGOBAY, Sadık Ahmet UYANIK, Savaş ALTINSOY, Reyhan POLAT, Jülide ERGİL

**Affiliations:** 1 Department of Anesthesiology and Reanimation, Ankara Etlik City Hospital, Ankara, Turkiye; 2 Department of Interventional Radiology, Ankara Etlik City Hospital, Ankara, Turkiye

**Keywords:** Quadratus lumborum block, percutaneous biliary drainage, nonoperating room anesthesia, capnography

## Abstract

**Background/aim:**

The aim of this study was to evaluate the effect of the quadratus lumborum block (QLB) on intraoperative opioid consumption in patients undergoing percutaneous biliary drainage (PBD).

**Materials and methods:**

A total of 57 patients who met the inclusion criteria were randomized into two groups: the sedation group (Group 1, n = 26) and the sedation with QLB group (Group 2, n = 26). The groups were compared in terms of intraoperative anesthetic consumption, vital parameters, postanesthesia care unit (PACU) recovery times, postoperative visual analog scale (VAS) scores, postoperative analgesic consumption, and clinician and patient satisfaction. The study was entered into the ClinicalTrials.gov database (Identifier: NCT06618781).

**Results:**

Demographic characteristics and anesthesia and surgical durations were comparable between the two groups (p > 0.05). Intraoperative total propofol and remifentanil consumption was significantly lower in Group 2 (p < 0.001). The incidence of complications was comparable between the groups (p > 0.05). Intraoperative hemodynamic stability was assessed by comparing heart rate (p > 0.05), systolic blood pressure (p = 0.043), diastolic blood pressure (p > 0.05), mean arterial pressure (p > 0.05), and end-tidal carbon dioxide (p > 0.05). Group 2 was found to be more stable in terms of systolic blood pressure. PACU recovery times were significantly shorter in Group 2 (p = 0.002). Postoperative VAS scores at the 2nd, 6th, 12th, and 24th hours, as well as the need for rescue analgesics, were significantly lower in Group 2 (p < 0.001). Clinician and patient satisfaction scores were significantly higher in Group 2 (p < 0.001).

**Conclusion:**

The current findings suggest that the incorporation of QLB into anesthetic management for PBD procedures may improve perioperative outcomes by reducing intraoperative opioid requirements without an associated increase in complication rates.

## 1. Introduction

Percutaneous transhepatic cholangiography and percutaneous biliary drainage (PBD) is a diagnostic and therapeutic procedure performed to achieve biliary decompression in patients with obstructive jaundice secondary to various etiologies, including choledocholithiasis, malignancy, and infection [1,2]. During PBD, both visceral and somatic pain may occur due to procedural manipulation and placement of a drainage catheter at the conclusion of the intervention [2,3]. Inadequate pain control may result in hemodynamic instability, reduced clinician and patient comfort, prolonged procedural duration with increased radiation exposure, and an increased requirement for additional analgesics such as opioids [4,5].

PBD may be performed under sedation or general anesthesia in the interventional radiology suite within the nonoperating room anesthesia (NORA) setting [6]. In recent years, regional anesthesia techniques have gained increasing attention, particularly in patients with significant comorbidities such as hepatic dysfunction, malignancy, and systemic diseases due to the potential adverse effects associated with general anesthesia in this high risk population [7,8].

Although regional anesthesia techniques such as the erector spinae plane block (ESPB) has been reported previously in the literature, the use of the quadratus lumborum block (QLB) in PBD has not been addressed. The QLB is an interfascial plane block that provides both visceral and somatic analgesia, covering a broad dermatomal distribution ranging approximately from T7 to L1 via spread through the thoracolumbar fascia (TLF), and it has proven analgesic efficacy in abdominal and pelvic surgeries [9]. In the QLB, the spread of local anesthetic within the TLF contributes to visceral analgesia through involvement of the sympathetic neuronal network and mechanoreceptors such as Ruffini and Pacinian corpuscles [10]. For these reasons, the QLB may offer certain potential advantages over other truncal block techniques for PBD [11]. Despite these theoretical benefits, however, the available evidence regarding the efficacy and safety of the QLB specifically for PBD remains limited.

This study evaluates the effect of the QLB on intraoperative opioid consumption in patients undergoing PBD.

## 2. Materials and methods

### 2.1. Ethical statement

This prospective randomized controlled study received approval from the Ethics Committee of Ankara Etlik City Hospital (Approval No: 0061). The trial was prospectively registered in the ClinicalTrials.gov database (NCT06618781), and participant recruitment was initiated only after completion of the registration process. All procedures were carried out in accordance with institutional ethical standards, the Helsinki Declaration, and similar ethical guidelines. The methodology, execution, and reporting of the study were carried out in accordance with international recommendations.

### 2.2. Study design

The study included patients aged 18–80 years with an American Society of Anesthesiologists (ASA) physical status classification of I–III and a body mass index (BMI) value of 18–40 kg/m^2^ who underwent PBD alone or in combination with the QLB under sedation between October 2024 and February 2025. The study was conducted in the interventional radiology unit of a tertiary hospital.

Exclusion criteria included age under 18 or over 80 years, ASA physical status classification of IV or higher, presence of bleeding diathesis, infection at the planned block site, BMI below 18 or above 40 kg/m^2^, unplanned emergency procedures, pregnancy, known allergy to opioids or local anesthetics, chronic pain syndromes, and cases in which a subxiphoid approach was planned for PBD. Patients were allocated to either the sedation group (Group 1) or the QLB block + sedation group (Group 2) using a randomized sealed opaque envelope method. To strengthen blinding and reduce performance bias, the anesthesiologist administering the preoperative regional anesthesia was different from the anesthesiologist responsible for intraoperative patient monitoring and management. In addition, to minimize detection bias, all postoperative outcome data, including visual analog scale (VAS) scores, patient satisfaction rates, and total analgesic consumption, were collected by an independent investigator who was strictly blinded to group allocation. Final statistical evaluation was performed using a per-protocol analysis strategy.

### 2.3. Interventions

All patients were informed about the study protocol and written informed consent was obtained. During the preoperative period, patients were divided into two groups:

Group 1: Sedation (n = 26)
Group 2: Lateral QLB with sedation (n = 26)

In the preoperative area of the interventional radiology unit, all patients underwent standard ASA monitoring, including noninvasive blood pressure measurement, electrocardiography, and peripheral oxygen saturation (SpO_2_). Intravenous (IV) access was established using a 20-gauge catheter. Patients allocated to the QLB group were provided with detailed information regarding the block procedure. In Group 2, the QLB was performed 30 min prior to the PBD procedure, with the patient in the left lateral decubitus position, under ultrasound guidance (5–1 MHz curvilinear probe) [12]. For lateral QLB (type 1), the probe was positioned at the T8–T10 level between the iliac crest and the subcostal margin and directed posteriorly and cranially to obtain detailed visualization of the transverse process and the anterolateral aspect of the vertebral body [13]. The characteristic shamrock sign formed by the psoas major, quadratus lumborum, and erector spinae muscles was identified [13]. Using a lateral approach, correct needle placement was confirmed by hydrodissection with 2 mL of normal saline at the lateral border of the quadratus lumborum muscle, at the point where the transversus abdominis muscle tapers. A total of 20 mL of local anesthetic solution comprising 14 mL of 0.5% bupivacaine and 6 mL of 2% lidocaine was injected into this plane [12]. This dose was chosen to provide a wide safety margin by remaining well below the weight-based toxic threshold (for bupivacaine 2 mg/kg, for lidocaine 4 mg/kg), which is clinically critical, especially for patients with potential hepatic impairment. The injectate was administered in divided doses of 5 mL with intermittent negative aspiration. Prior to the procedure, the success of the block was confirmed by a pinprick test (loss of cold and pain sensation) in the thoracolumbar dermatomes. The QLB block procedure was performed by a single experienced anesthesiologist. Following the block procedure, patients were monitored again in the preoperative area. All patients received 2 mg of IV midazolam 5 min prior to transfer to the procedure room. After application of standard ASA monitoring in the procedure room, anesthesia was induced with 0.5 mg/kg propofol and 0.5 μg/kg fentanyl. Propofol and remifentanil infusions were titrated to maintain bispectral index values between 60 and 80 and ensure adequate analgesia, defined by a VAS score of <4. Remifentanil infusion was initiated at a dose of 0.1–1 μg/kg/min and adjusted according to clinical response [14,15]. End-tidal carbon dioxide (EtCO_2_) was monitored using a procedural oxygen mask (POM Procedural Oxygen Mask, Stryker Corporation, Portage, MI, USA), and supplemental oxygen was administered at 4 L/min. Vital parameters were recorded throughout the procedure. Intraoperative mean arterial pressure (MAP) of <65 mmHg was defined as hypotension, heart rate (HR) of <60 beats/min as bradycardia, and SpO_2_ of <90% as desaturation [16,17]. Postoperative nausea and vomiting (PONV) were recorded if patients reported nausea or experienced at least one episode of vomiting during the observation period. All patients received 1 g of IV paracetamol postoperatively, and 50 mg of dexketoprofen was administered as rescue analgesia when the VAS score reached >4. In the postanesthesia care unit (PACU), recovery time was recorded in minutes when the modified Aldrete score exceeded 9. Postoperative VAS scores were recorded at the 2nd, 6th, 12th, and 24th hours. Clinician and patient satisfaction were evaluated as insufficient (0), moderate (1), good (2), or perfect (3). Percutaneous transhepatic cholangiography and PBD were performed for all patients according to the same institutional protocol.

### 2.4. Outcomes

The study’s primary outcome was total intraoperative remifentanil consumption. Secondary outcomes included total propofol consumption, postoperative VAS scores, PACU recovery times, procedural hemodynamic stability, and patient and clinician satisfaction.

### 2.5. Statistical analysis

Sample size was calculated using G*Power (Heinrich Heine University Düsseldorf, Düsseldorf, Germany) based on preliminary data on mean total remifentanil consumption. Mean total remifentanil consumption was 0.43 ± 0.15 mg in the QLB group and 0.71 ± 0.54 mg in the control group (α = 0.05, power = 80%, effect size = 0.71). According to this calculation, at least 52 participants were required. Therefore, to account for a 10% potential participant dropout rate, 57 patients were enrolled.

The normality of the data was assessed using the Shapiro–Wilk test, visual analysis of histograms, and Q–Q plots. The chi-square or Fisher exact test was used to compare categorical variables. Continuous variables were compared using the independent-samples t-test for normally distributed data and the Mann–Whitney U test for nonnormally distributed data. Repeated hemodynamic measurements were analyzed using linear mixed-effects models (LMMs) to account for time, group, and group × time interactions. Bonferroni correction was applied to multiple comparisons to minimize type I error. Effect sizes were estimated using Cohen d values with 95% confidence intervals (CIs) for parametric variables and r values for nonparametric variables, while phi and Cramer V coefficients were used for categorical variables, as appropriate. For Cohen d values, results of 0.20–0.49 were considered to indicate small, 0.50–0.79 medium, and ≥0.80 large effect sizes. For r, phi coefficient, and Cramer V values, 0.10–0.29 indicated small, 0.30–0.49 medium, and ≥0.50 large effect sizes. For partial η^2^, values of 0.01–0.05 indicated small, 0.06–0.13 medium, and ≥0.14 large effect sizes. All statistical analyses were performed using IBM SPSS Statistics 26.0 (IBM Corp., Armonk, NY, USA), and values of p < 0.05 were considered statistically significant.

## 3. Results

In this prospective randomized controlled study conducted between October 2024 and February 2025, the perioperative effects of sedation alone and sedation combined with QLB were compared in patients undergoing PBD. Initially, 57 patients who met the eligibility criteria were enrolled. However, two patients in Group 1 were withdrawn from the study because they required a switch to general anesthesia due to surgical complications. In Group 2, one patient was excluded because the PBD procedure was cancelled, and two patients were excluded due to inadequate and unsuccessful ultrasound imaging. Thus, data from 52 patients were evaluated using per-protocol analysis (Figure).

No statistically significant differences were observed between the groups for demographic or intraprocedural data, comorbidities, anesthesia duration, or surgical duration (p > 0.05; Tables 1 and 2). In addition, effect sizes for all of these variables were small or negligible (Tables 1 and 2).

However, intraoperative total propofol consumption was significantly lower in Group 2 than in Group 1 (43.46 ± 15.67 mg vs. 105 ± 51.94 mg, p < 0.001). Similarly, total remifentanil consumption was significantly lower in Group 2 [0 (interquartile range: 0–0.10) mg vs. 0.75 (interquartile range: 0.39–1.26) mg, p < 0.001] (Table 2). The mean difference in total propofol consumption between the groups was 61.54 mg (95% CI: 41.28–81.50 mg), indicating a high effect size (Cohen d = −1.60). Total remifentanil intake also had a high effect size (r = 0.75; Table 2).

No statistically significant differences were observed between the groups with respect to intraoperative or postoperative complications (Tables 2 and 3). Comparison of PACU recovery times demonstrated significantly shorter recovery durations in Group 2 than in Group 1 (22.84 ± 3.37 min vs. 37.73 ± 5.89 min, p = 0.002; Table 3). The mean difference between the groups was 14.88 min (95% CI: 12.21–17.56 min), with a large effect size (Cohen d = 3.10).

In Group 2, clinical physician and patient satisfaction scores were significantly higher compared to Group 1. Clinical physician satisfaction scores were 2 (2–2) in Group 1 and 2.5 (2–3) in Group 2 (p < 0.001), with a large effect size (r = 0.585). Similarly, patient satisfaction scores were significantly higher in Group 2 compared to Group 1 [3 (2–3) and 1 (1–2), respectively; p < 0.001], corresponding to a large effect size (r = 0.740; Table 3). The need for postoperative salvage analgesics was significantly lower in Group 2 (p < 0.001), with a large effect size (phi = −0.583). The time to first analgesic need was significantly longer in Group 2 [6 (5–12) h vs. 2 (2–4) h, p < 0.001], and the effect size was moderate to large (r = 0.55; Table 3).

LMMs were used to compare repeated hemodynamic measurements [systolic blood pressure (SBP), diastolic blood pressure (DBP), MAP, HR, and EtCO_2_] between the groups over time. “Patient ID” was included in the model as a random intercept to control for individual differences in baseline measurements and Bonferroni correction was applied for multiple comparisons. Examining the curves of change in hemodynamic parameters, a statistically significant difference was found for SBP between groups and times (p = 0.043, partial η^2^ = 0.12; Table 4); however, no statistically significant differences were found for MAP, DBP, HR, and EtCO_2_ (p > 0.05; Table 4). When the estimated marginal means for SBP were evaluated, Group 2 demonstrated strict systolic stability, remaining within a narrow range throughout the procedure (125.4–122.6 mmHg). In contrast, Group 1 started the procedure with higher values (136.4 mmHg) and exhibited broader hemodynamic fluctuations, with a marked hypotensive trend observed at 45 min (119.3 mmHg).

Changes in VAS scores at the 2nd, 6th, 12th, and 24th hours postoperatively and differences between the groups were analyzed using LMMs and Bonferroni correction. The analysis revealed a statistically significant difference between groups and times in terms of the course of postoperative pain (p < 0.001, partial η^2^ = 0.68; Table 5). A large effect size was observed for the group × time interaction (partial η^2^ = 0.68). Analysis of the estimated marginal means demonstrated that patients in Group 2 had substantially lower pain scores at the postoperative 2nd hour 2 (3.19 ± 0.19) compared to Group 1 (6.15 ± 0.19), and this analgesic superiority was maintained throughout the 24-h follow-up period. While pain scores in Group 1 decreased to only 3.06 at the postoperative 24th hour, the corresponding value in Group 2 declined to 1.27 (Table 5).

## 4. Discussion

This study compared sedation alone with sedation combined with the QLB in patients undergoing PBD. The findings suggest that the QLB was associated with reduced intraoperative total opioid consumption, faster postoperative recovery, improved clinician and patient satisfaction, and lower postoperative VAS scores at the 2nd, 6th, 12th, and 24th hours, without a significant increase in complication rates. Although favorable trends in intraoperative hemodynamic parameters were also observed, no statistically significant differences were identified between the groups.

The QLB extends into the paravertebral and epidural spaces, providing analgesia in the T7–L1 dermatomes [18,19]. The analgesic efficacy of the QLB has also been demonstrated in studies involving abdominal, pelvic, peritoneal, and orthopedic surgeries [13,20,21]. In interventional radiology, various regional anesthesia techniques have been described for percutaneous biliary tract interventions and hepatic procedures, including the paravertebral block (PVB), ESPB, aortorenal plexus plane block, superior hypogastric plexus plane block, and QLB [22–24]. However, studies specifically evaluating the efficacy of the QLB for PBD have been relatively limited compared to other regional block techniques [22–24].

Regional anesthesia techniques are gaining increasing attention due to their potential to reduce opioid requirements in abdominal and hepatobiliary procedures. In a study in which ESPB was performed for liver ablation, it was reported that 74% of participants did not require deep sedation or general anesthesia [22]. In a study by Culp et al., PVB with sedation was shown to be effective in pain control for biliary drainage [25]. However, considering the close anatomical relationship of the ESPB and PVB with neuraxial structures, as well as the potential risks of complications due to their proximity to the peritoneum and pleura, the QLB stands out as an alternative regional anesthesia technique due to its more prominent anatomical landmarks and ease of application. From among the four different approaches possible for the QLB, we selected the lateral QLB (type 1) because it is relatively superficial, easy to perform, and associated with a low complication rate [26]. A published case report indicated that the QLB was successfully used as the primary anesthesia technique in a high-comorbidity patient undergoing emergency colostomy; the procedure was completed without complications and without the need for opioids [27]. Our current findings suggest that the QLB significantly reduces intraoperative total remifentanil consumption for PBD.

Regional anesthesia techniques reduce opioid requirements, contributing to a lower incidence of opioid-related side effects such as sedation, nausea and vomiting, respiratory depression, and hemodynamic instability, thus positively impacting recovery and discharge [6,9,19,28]. Similar to previous studies using the ESPB and PVB for hepatobiliary interventions [22,24,25], our study observed that the QLB for PBD resulted in shorter PACU recovery times, but hospital discharge could not be evaluated. In a study comparing the QLB, ESPB, and a control group, it was shown that the QLB and ESPB contributed to recovery [28]. However, it should not be forgotten that alongside the advantages provided by regional techniques, there are also serious and significant side effects, such as local anesthetic systemic toxicity (LAST), although they are rare. While our study did not show a significant difference in terms of simpler complications such as bradycardia, hypotension, desaturation, and PONV with the QLB, rare complications such as LAST may have been missed due to our limited sample size.

In a study conducted by Kwak et al., the QLB was shown to provide effective postoperative analgesia in patients undergoing laparoscopic nephrectomy, while another study reported that the QLB significantly reduced VAS scores after total hysterectomy [29,30]. Similar to findings reported in abdominal and pelvic surgeries, our study demonstrated that the QLB significantly reduced postoperative VAS scores during the first 24 h after PBD and maintained lower pain scores throughout the follow-up period. Furthermore, the need for postoperative salvage analgesics was observed to be lower in the QLB group; this may be related to lower VAS scores.

When physician and patient satisfaction were compared between the groups, higher satisfaction rates were observed in the QLB group. Higher patient satisfaction may be associated with lower postoperative VAS scores. Although physician satisfaction is inherently a subjective parameter, we attributed this difference to the uninterrupted continuation of the PBD procedure under more stable anesthetic conditions. Despite the better outcomes observed in terms of VAS scores, analgesic consumption, and satisfaction, it should be kept in mind that different results may occur due to factors such as variability between clinicians, differences in the number of puncture attempts required for biliary access, individual patient differences, comorbidities, and the heterogeneous underlying etiologies of the patients.

Several studies have shown that sedation is more commonly preferred in the NORA setting because of the potential complications of general anesthesia and the challenges associated with airway management [4,6,31]. In a study by Ebert, the combination of propofol with opioids such as remifentanil was shown to contribute to analgesia during sedation due to the rapid onset and potent hypnotic properties of propofol [32]. Furthermore, multimodal regimens including combinations of intravenous agents have been recommended to reduce neuroendocrine, autonomic, and stress responses [4]. In line with the literature, we implemented a multimodal anesthesia strategy involving the QLB together with propofol and remifentanil.

Surgical stress and pain lead to increased neuroendocrine activation and catecholamine release. In patients with high comorbidity and limited functional reserve, these physiological changes have been associated with hemodynamic instability and increased perioperative complications [33]. In a study by Rathod et al., the QLB was shown to reduce the hemodynamic response and lower neuroendocrine stress in pediatric patients undergoing open pyeloplasty [34]. Similarly, Jiang et al. demonstrated that the QLB provided effective analgesia in the early postoperative period, improved the hemodynamic response, and enhanced respiratory stability in patients undergoing colorectal surgery [33]. In our study, while a statistically significant difference in systolic blood pressure was observed between the groups, no differences were observed in other parameters. The intermittent and task-dependent nature of nociceptive stimuli during PBD, combined with interindividual variability in the sedative response, may have limited the ability to detect intergroup differences in hemodynamic variables. Therefore, the isolated SBP effect may serve as a more specific indicator of analgesic modulation rather than a global hemodynamic change. However, despite the significant difference in total anesthetic consumption between groups, this result regarding hemodynamic parameters may have also stemmed from our limited sample size and interindividual variability.

Studies have shown that, during sedation with spontaneous respiration, clinical assessment using pulse oximetry and observation of chest wall movements is insufficient for early detection of hypoxia, whereas the use of capnography reduces the incidence of hypoxia by approximately 50% [35,36]. For this reason, we used an oxygen mask with continuous capnography monitoring (POM Procedural Oxygen Mask) in our study. This mask, which includes an integrated capnography system, allowed early detection of inadequate ventilation and was selected to help prevent potential respiratory complications. Although desaturation was observed in one patient, it was attributed to peripheral vasoconstriction due to hypotension rather than true ventilation impairment and did not require airway intervention. We therefore suggest that routine use of capnography during sedation in NORA procedures such as PBD may help reduce airway-related complications [37].

This study has several limitations. Patients were only monitored for 24 h; therefore, the long-term effects of QLB could not be assessed. Furthermore, only QLB was applied for PBD; other truncal block techniques were not included in the comparison. Because this was a single-center study, its reproducibility may be limited in centers with less experienced practitioners. Postoperative VAS scores and clinician and patient satisfaction assessments are subjective measures by nature. Individual variability in pain perception was not considered, which may have led to performance bias. Moreover, due to the nature of the study, a protocol-bound approach was adopted. This may introduce a risk of selection bias and potentially increase the treatment effect.

Our findings demonstrated that the use of the QLB during PBD was associated with reduced intraoperative opioid requirements, faster recovery, lower postoperative pain scores, and higher patient satisfaction. These results suggest that the QLB may constitute an effective analgesic strategy for NORA procedures such as PBD, particularly in patients with significant comorbidities. Further prospective studies with larger sample sizes and comparative designs are warranted to better clarify its role in this setting.

## Figures and Tables

**Figure:**
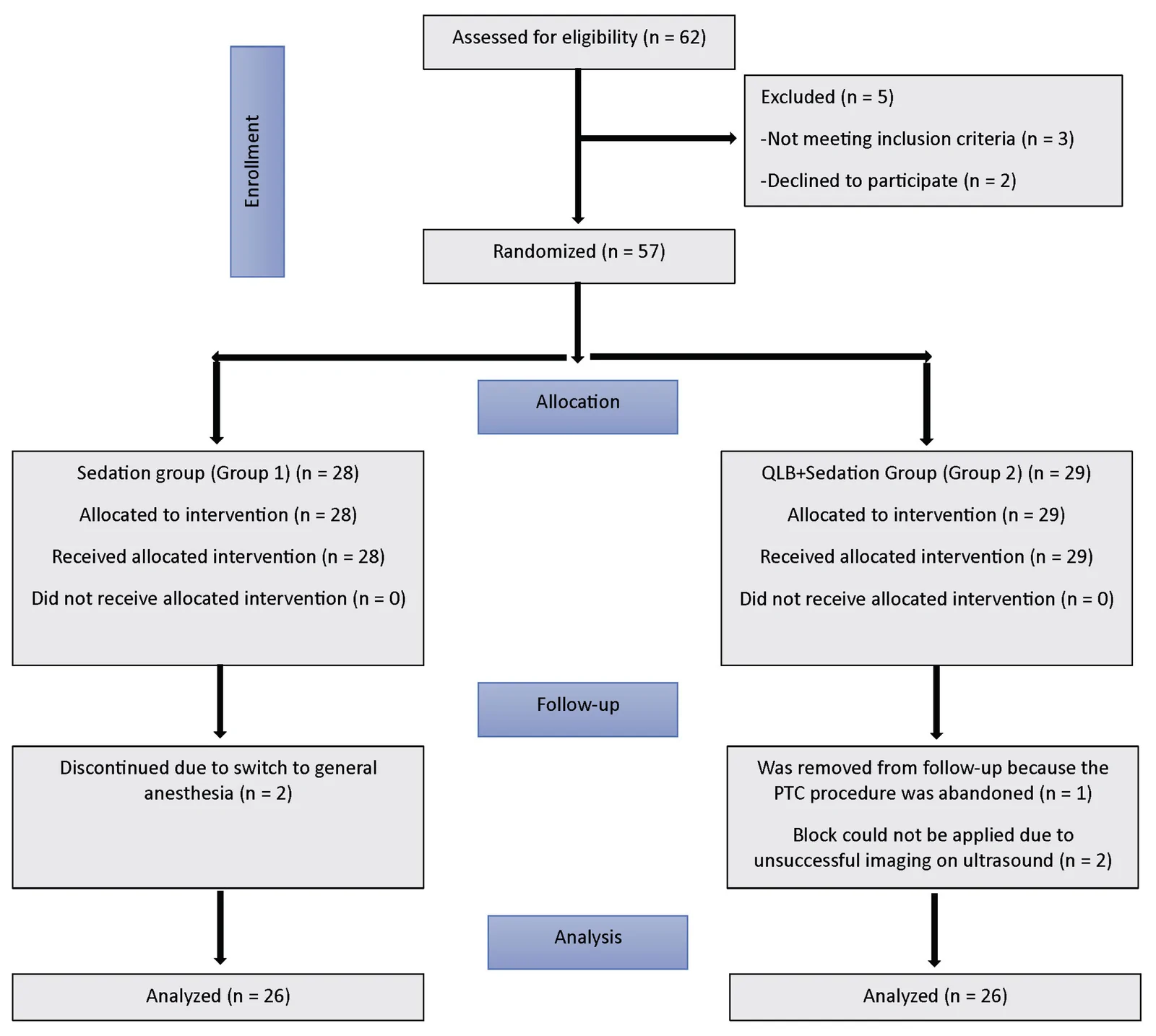
CONSORT flow diagram.

**Table 1:** Demographic data of patients.

	Group 1 (sedation) (n = 26)	Group 2 (QLB + sedation) (n = 26)	p*	95% CI for difference	Effect size**
Age (year)	64.42 ± 13.18	62.62 ± 12.94	0.620^1^	[−5.17, 8.61]	d = 0.14
BMI	28.08 ± 2.73	28.98 ± 4.16	0.361^1^	[−2.94, 0.94]	d = 0.26
Sex			1.000		Phi = −0.038
Female	13 (50)	14 (53.8)			
Male	13 (50)	12 (46.2)			
ASA Classification			1.000		Cramer’s V = 0.044
*I*	0	0			
*II*	7 (26.9)	6 (23.1)			
*III*	19 (73.1)	20 (76.9)			
*IV*	0	0			
Comorbidity			1.000		Phi = 0.00
*Yes*	25 (96.2)	25 (96.2)			
*No*	1 (3.8)	1 (3.8)			

Categorical variables are presented as n (%) and continuous variables as mean ± standard deviation.

1 Independent-sample t-test.

BMI: Body mass index. ASA: American Society of Anesthesia.

* p < 0.05 was considered statistically significant.

** Effect sizes are reported as Cohen d, r, phi, partial η^2^, or Cramer V values, as appropriate.

**Table 2:** Intraoperative data.

	Group 1 (sedation) (n = 26)	Group 2 (QLB + sedation) (n = 26)	p*	95% CI for difference	Effect size**
Propofol (total consumption) (mg)	105 ± 51.94	43.46 ± 15.67	**<0.001** 1	[41.28, 81.50]	d = −1.60
Remifentanil (total consumption) (mg)	0.75 (0.39–1.26)	0 (0–0.10)	**<0.001** 2		r = 0.75
Anesthesia duration (min)	35 (32–45.75)	39.50 (34–45.25)	0.900^2^		r = 0.09
Surgical duration (min)	30 (26.50–40.50)	33 (27.75–40)	0.831^2^		r = 0.05
Complication			0.522		Phi = −0.133
*No*	18 (69.2)	21 (80.8)			
*Yes*	8 (30.8)	5 (19.2)			
	Bradycardia 2 (7.7)	Bradycardia 2 (7.7)			
	Hypotension 4 (15.4)	Hypotension 2 (7.7)			
	Hypotension + Bradycadia 2 (7.7)	Desaturation + Hypotension: 1 (3.8)			

Categorical variables are presented as n (%) and continuous variables as mean ± standard deviation or median [interquartile range (IQR): 25th–75th percentiles].

1 Independent-sample t-test.

2 Mann–Whitney U test.

* Statistically significant p-values (p < 0.05) are shown in bold.

** Effect sizes are reported as Cohen d, r, phi, partial η^2^, or Cramer V values, as appropriate.

**Table 3:** Postoperative data.

	Group 1 (sedation) (n = 26)	Group 2 (QLB + sedation) (n = 26)	p*	95% CI for difference	Effect size**
PONV			0.465		Phi = −0.152
*No*	20 (76.9)	23 (88.5)			
*Yes*	6 (23.1)	3 (11.5)			
PACU recovery time (min)	37.73 ± 5.89	22.84 ± 3.37	**0.002** 1	[12.21, 17.56]	d = 3.10
Clinician satisfaction***	2 (2–2)	2.5 (2–3)	**<0.001** 2		r = 0.585
Patient satisfaction***	1(1–2)	3 (2–3)	**<0.001** 2		r = 0.740
PO rescue analgesic			**<0.001**		Phi = −0.583
* No*	1 (3.8)	15 (57.7)			
* Yes*	25 (96.2)	11 (42.3)			
Analgesia time (hours)	2 (2–4)	6 (5–12)	**<0.001** 2		r = 0.55

Categorical variables are presented as n (%) and continuous variables as mean ± standard deviation or median [interquartile range (IQR) 25th–75th percentiles].

1 Independent-samples t-test.

2 Mann–Whitney U test.

PONV: Postoperative nausea and vomiting. PACU: Postanesthetic care unit. PO: Postoperative.

* Statistically significant p-values (p < 0.05) are shown in bold.

** Effect sizes are reported as Cohen d, r, phi, partial η^2^, or Cramer V values, as appropriate.

*** Satisfaction scores were coded from 0 to 3 (0 = insufficient, 1 = moderate, 2 = good, 3 = perfect).

**Table 4:** Estimated marginal means of hemodynamic and respiratory parameters during the procedures.

Parameter / time	Group 1 (sedation) (n = 26) Mean ± SE [95% CI]	Group 2 (QLB + sedation) (n = 26) Mean ± SE [95% CI]	Interaction p-value*	Effect size** (partial η2)
Systolic blood pressure (mmHg)			**0.043**	0.12
Baseline (0 min)	136.4 ± 3.7 [129.1, 143.7]	125.4 ± 3.7 [118.1, 132.7]		
5 min	127.7 ± 3.7 [120.4, 135.0]	125.0 ± 3.7 [117.7, 132.3]		
15 min	131.6 ± 3.7 [124.3, 138.9]	123.4 ± 3.7 [116.1, 130.7]		
30 min	130.7 ± 3.8 [123.3, 138.1]	122.6 ± 3.8 [115.2, 130.0]		
45 min	119.3 ± 5.0 [109.5, 129.1]	123.0 ± 4.8 [113.6, 132.4]		
60 min	134.3 ± 7.5 [119.6, 149.0]	N/A		
Mean arterial blood pressure (mmHg)			0.181	0.06
Baseline (0 min)	96.3 ± 3.0 [90.4, 102.2]	88.4 ± 3.0 [82.5, 94.3]		
5 min	90.8 ± 3.0 [84.9, 96.7]	89.0 ± 3.0 [83.1, 94.9]		
15 min	91.4 ± 3.0 [85.5, 97.3]	86.8 ± 3.0 [80.9, 92.7]		
30 min	93.4 ± 3.2 [87.1, 99.7]	85.0 ± 3.1 [78.8, 91.2]		
45 min	84.1 ± 4.4 [75.5, 92.7]	84.3 ± 4.2 [76.1, 92.5]		
60 min	95.9 ± 6.8 [82.6, 109.2]	N/A		
Diastolic blood pressure (mmHg)			0.502	0.03
Baseline (0 min)	76.2 ± 2.8 [70.7, 81.7]	69.7 ± 2.8 [64.2, 75.2]		
5 min	71.8 ± 2.8 [66.3, 77.3]	69.8 ± 2.8 [64.3, 75.3]		
15 min	73.1 ± 2.8 [67.6, 78.6]	68.4 ± 2.8 [62.9, 73.9]		
30 min	73.9 ± 2.9 [68.2, 79.6]	68.1 ± 2.9 [62.4, 73.8]		
45 min	66.9 ± 3.9 [59.3, 74.5]	64.9 ± 3.7 [57.6, 72.2]		
60 min	77.9 ± 5.9 [66.4, 89.4]	N/A		
Heart rate (bpm)			0.068	0.09
Baseline (0 min)	78.4 ± 3.0 [72.5, 84.3]	82.4 ± 3.0 [76.5, 88.3]		
5 min	83.3 ± 3.0 [77.4, 89.2]	81.3 ± 3.0 [75.4, 87.2]		
15 min	83.2 ± 3.0 [77.3, 89.1]	79.2 ± 3.0 [73.3, 85.1]		
30 min	82.4 ± 3.1 [76.3, 88.5]	79.5 ± 3.1 [73.4, 85.6]		
45 min	74.7 ± 4.3 [66.3, 83.1]	77.7 ± 4.1 [69.7, 85.7]		
60 min	85.6 ± 6.3 [73.3, 97.9]	N/A		
End-tidal carbon dioxide (mmHg)			0.223	0.04
Baseline (0 min)	31.5 ± 1.0 [29.5, 33.5]	30.3 ± 1.0 [28.3, 32.3]		
5 min	29.7 ± 1.0 [27.7, 31.7]	30.2 ± 1.0 [28.2, 32.2]		
15 min	30.5 ± 1.0 [28.5, 32.5]	30.0 ± 1.0 [28.0, 32.0]		
30 min	31.9 ± 1.0 [29.9, 33.9]	30.4 ± 1.0 [28.4, 32.4]		
45 min	31.1 ± 1.3 [28.6, 33.6]	31.1 ± 1.3 [28.6, 33.6]		
60 min	29.4 ± 1.9 [25.7, 33.1]	N/A		

Data are presented as estimated marginal means ± standard errors derived from the linear mixed-effects models (LMMs). Bonferroni correction was applied for multiple comparisons. N/A: Not applicable (procedure completed before this time point).

SE: Standard error. QLB: Quadratus lumborum block.

* p < 0.05 (shown in bold) was considered statistically significant.

** Effect sizes are reported as Cohen d, r, phi, partial η^2^, or Cramer V values, as appropriate. Since no participant in Group 2 had a surgical duration exceeding 60 min, the 60-min data were not included in the LMM analysis.

**Table 5:** Estimated marginal means of postoperative VAS scores.

Time point	Group 1 (sedation) (n = 26)	Group 2 (QLB + sedation) (n = 26)	Interaction p-value*	Effect size** (partial η2)
PO 2nd hour	6.15 ± 0.19 [5.77, 6.53]	3.19 ± 0.19 [2.81, 3.57]	**<0.001** *	0.68
PO 6th hour	4.92 ± 0.19 [4.54, 5.30]	2.73 ± 0.19 [2.35, 3.11]		
PO 12th hour	4.27 ± 0.19 [3.89, 4.65]	2.04 ± 0.19 [1.66, 2.42]		
PO 24th hour	3.06 ± 0.20 [2.66, 3.46]	1.27 ± 0.19 [0.89, 1.65]		

VAS: Visual analog scale. SE: Standard error. QLB: Quadratus lumborum block. Data are presented as estimated marginal means ± standard errors derived from linear mixed-effects models. Bonferroni correction was applied for multiple comparisons. PO: Postoperative.

* p < 0.05 (shown in bold) was considered statistically significant.

** Effect sizes are reported as Cohen d, r, phi, partial η^2^, or Cramer V values, as appropriate.
